# Treatment of Peritoneal Dissemination in Stomach Cancer Patients With Cytoreductive Surgery and Hyperthermic Intraperitoneal Chemotherapy (HIPEC): Rationale and Design of the PERISCOPE Study

**DOI:** 10.2196/resprot.7790

**Published:** 2017-07-13

**Authors:** Rosa T van der Kaaij, Hidde JW Braam, Henk Boot, Maartje Los, Annemieke Cats, Cecile Grootscholten, Jan HM Schellens, Arend GJ Aalbers, Alwin DR Huitema, Catherijne AJ Knibbe, Djamila Boerma, Marinus J Wiezer, Bert van Ramshorst, Johanna W van Sandick

**Affiliations:** ^1^ The Netherlands Cancer Institute – Antoni van Leeuwenhoek Hospital Department of Surgical Oncology Amsterdam Netherlands; ^2^ St. Antonius Hospital Department of Surgery Nieuwegein Netherlands; ^3^ The Netherlands Cancer Institute – Antoni van Leeuwenhoek Hospital Department of Gastroenterology Amsterdam Netherlands; ^4^ St. Antonius Hospital Department of Medical Oncology Nieuwegein Netherlands; ^5^ The Netherlands Cancer Institute – Antoni van Leeuwenhoek Hospital Department of Medical Oncology Amsterdam Netherlands; ^6^ The Netherlands Cancer Institute – Antoni van Leeuwenhoek Hospital Department of Clinical Pharmacology Amsterdam Netherlands; ^7^ The Netherlands Cancer Institute – Antoni van Leeuwenhoek Hospital Department of Pharmacy and Pharmacology Amsterdam Netherlands; ^8^ University Medical Center Utrecht Department of Clinical Pharmacy Utrecht Netherlands; ^9^ St. Antonius Hospital Department of Pharmacology Nieuwegein Netherlands

**Keywords:** gastric cancer, peritoneal carcinomatosis, gastrectomy, cytoreduction, HIPEC, hyperthermic intraperitoneal chemotherapy, oxaliplatin, docetaxel

## Abstract

**Background:**

Patients with gastric cancer and peritoneal carcinomatosis have a very poor prognosis; median survival is 3 to 4 months. Palliative systemic chemotherapy is currently the only treatment available in the Netherlands. Intraoperative hyperthermic intraperitoneal chemotherapy (HIPEC) has an established role in the treatment of peritoneal carcinomatosis originating from colorectal cancer, appendiceal cancer, and pseudomyxoma peritonei; its role in gastric cancer is uncertain. Currently, there is no consensus on the choice of chemotherapeutic agents used in HIPEC for gastric cancer.

**Objective:**

The main objectives of this study are (1) to investigate the safety, tolerability, and feasibility of gastrectomy combined with cytoreductive surgery and HIPEC after systemic chemotherapy, as a primary treatment option for patients with advanced gastric cancer with tumor positive peritoneal cytology and/or limited peritoneal carcinomatosis; and (2) to determine the maximum tolerated dose (MTD) of intraperitoneal docetaxel in combination with a fixed dose of intraperitoneal oxaliplatin.

**Methods:**

The PERISCOPE study is a multicenter, open label, phase I-II dose-escalation study. The MTD of docetaxel will be studied using a 3+3 design. Patients with locally advanced (cT3-cT4) gastric adenocarcinoma are eligible for inclusion if the primary gastric tumor is considered resectable, tumor positive peritoneal cytology and/or limited peritoneal carcinomatosis is confirmed by diagnostic laparoscopy/ laparotomy, and prior systemic chemotherapy was without disease progression. At laparotomy, cytoreductive surgery (complete removal of all macroscopically visible tumor deposits) and a total or partial gastrectomy with a D2 lymph node dissection is performed. An open HIPEC technique is used with 460mg/m2 hyperthermic oxaliplatin for 30 minutes (41°C to 42°C) followed by normothermic docetaxel for 90 minutes (37°C) in a dose that will be escalated per 3 patients (0, 50, 75, 100, 125, 150 mg/m2). The primary endpoint is treatment related toxicity.

**Results:**

Patient accrual is ongoing and the first results are expected in 2017.

**Conclusions:**

The PERISCOPE study will determine the safety, tolerability, and feasibility of gastrectomy combined with cytoreduction and HIPEC using oxaliplatin in combination with docetaxel after systemic chemotherapy as primary treatment option for gastric cancer patients with tumor positive peritoneal cytology and/or limited peritoneal carcinomatosis. This study will provide pharmacokinetic data on the intraperitoneal administration of oxaliplatin and docetaxel, including the MTD of intraperitoneal-administered docetaxel. These data are a prerequisite for the safe conduct of future HIPEC studies in patients with gastric cancer.

**Trial Registration:**

Netherlands Trial Registration (NTR): NTR4250; http://www.trialregister.nl/trialreg/admin/ rctview.asp?TC=4250 (Archived by WebCite at http://www.webcitation.org/6rWJONgkt)

## Introduction

Patients with advanced gastric cancer have a poor prognosis. The 5-year survival rate is around 30%, even after potentially curative treatment [1-3]. In approximately 15% of patients the peritoneum is synchronously affected at diagnosis [4]. Patients with gastric cancer and peritoneal carcinomatosis have a very poor prognosis with a median survival of about 3 to 4 months without treatment [5]. It has been proposed that extended resection consisting of cytoreductive surgery (ie, complete removal of all macroscopically visible tumor deposits) and gastrectomy, combined with intraperitoneal chemotherapy, could improve survival in patients with peritoneal carcinomatosis from gastric cancer [6]. The limited permeability of the peritoneal plasma barrier allows the delivery of high concentrations of chemotherapeutic drugs directly into the peritoneal cavity without the danger of high plasma concentrations and subsequent systemic toxicity [7].

Intraoperative hyperthermic intraperitoneal chemotherapy (HIPEC) has an established role in the treatment of peritoneal carcinomatosis originating from colorectal cancer, appendiceal cancer, and pseudomyxoma peritonei [7,8]; however, the role of HIPEC in the treatment of gastric cancer with peritoneal carcinomatosis is uncertain. A systematic review showed that good quality evidence is limited as many studies included heterogeneous patient populations and differed in type, timing, method, and duration of drug delivery [9]. Better-designed studies showed longer survival of patients receiving intraperitoneal chemotherapy and cytoreductive surgery than those treated with surgery alone. Currently, various intraperitoneal chemotherapeutic drugs are used in gastric cancer but the best and the most suitable regimen is unknown [10]. An important issue which needs to be addressed is the choice of intraperitoneal chemotherapy and the most effective dose.

This study aims to evaluate the safety, tolerability, and feasibility of gastrectomy combined with cytoreductive surgery and HIPEC with oxaliplatin and docetaxel after systemic chemotherapy, as primary treatment for gastric cancer patients with tumor positive peritoneal cytology and/or limited peritoneal carcinomatosis.

## Methods

### Objectives

#### Primary Objective

The primary objective of this study is to investigate the safety, tolerability, and feasibility of gastrectomy combined with cytoreductive surgery and HIPEC after systemic chemotherapy, as a primary treatment option for advanced gastric cancer with tumor positive peritoneal cytology and/or limited peritoneal carcinomatosis.

#### Secondary Objectives

The secondary objectives are (1) to determine the maximum tolerated dose (MTD) of intraperitoneal docetaxel that can be given in combination with a fixed dose of oxaliplatin in patients with gastric cancer undergoing a gastrectomy combined with cytoreductive surgery and HIPEC after systemic chemotherapy; (2) to investigate the pharmacokinetics of intraoperative intraperitoneal-administered oxaliplatin and docetaxel; and (3) to determine the 2-year disease-free and overall survival of patients with advanced gastric cancer with tumor positive peritoneal cytology and/or limited peritoneal carcinomatosis, treated with gastrectomy, cytoreductive surgery and HIPEC.

### Study Population

Patients, 18 years or older, with biopsy proven surgically resectable (cT3-cT4, any N) gastric adenocarcinoma with tumor positive peritoneal cytology and/or limited peritoneal carcinomatosis are eligible for participation. Patients have to be treated with systemic chemotherapy, preferably 3 to 4 courses, with the last course ending within 8 weeks prior to inclusion. Accepted neoadjuvant chemotherapy regimens generally consist of a platinum drug combined with a fluoropyrimidine. In addition, an anthracycline or taxane may have been added according to local protocol. Examples of accepted chemotherapy regimens are (1) docetaxel with oxaliplatin and capecitabine (DOC); (2) docetaxel with cisplatin and 5-fluorouracil (5-FU; DCF); (3) epirucibin with cisplatin and capecitabine (ECC); and (4) epirucibin with oxaliplatin and capecitabine (EOC). Progressive disease under systemic chemotherapy precludes inclusion. Patients with metachronous peritoneal carcinomatosis, distant metastases, or recurrent gastric cancer will not be eligible for the current study. An overview of the inclusion and exclusion criteria is shown in Textbox 1 [11].

**Inclusion and exclusion criteria for the PERISCOPE study.**

Criteria

- Inclusion criteria
  - Age 18 years or older
  - World Health Organization (WHO) performance status 0 to 2
  - American Society of Anesthesiologists classification I to III
  - Biopsy proven adenocarcinoma of the stomach (also tumors at the esophagogastric junction with the bulk located in the stomach for which the intended surgical treatment is a gastric resection, not a resection of the esophagus and cardia)
  - T3-T4 tumor according to the 7th edition of the tumor, node, metastasis (TNM) classification system [11]
  - Tumor positive peritoneal cytology and/or peritoneal carcinomatosis limited to the upper abdominal cavity (above the transverse colon) and/or at the most at one location in the lower abdominal cavity (eg, Douglas’ pouch, ovarian metastasis, Sister Mary Joseph nodule) confirmed by diagnostic laparoscopy or laparotomy
  - Treated with neoadjuvant systemic chemotherapy (last course ending within 8 before inclusion)
  - Adequate bone marrow, hepatic, and renal function. Acceptable laboratory values at inclusion:
    - Absolute neutrophil count greater than or equal to 1.5 x 10^9^/L
    - Platelet count greater than or equal to 100 x 10^9^/L
    - Serum bilirubin less than or equal to 1.5 times the upper limit of normal (ULN) and alanine transaminase and aspartate transaminase less than or equal to 2.5 times ULN
    - Creatinine clearance greater than or equal to 50 ml/min (measured or calculated by Cockcroft-Gault formula)
  - Negative pregnancy test (urine/serum) for female patients with childbearing potential
  - Life expectancy 3 months or greater
  - Able and willing to undergo blood sampling for pharmacokinetics
  - Written informed consent
- Exclusion criteria
  - Distant metastases (eg, liver, lung, para-aortic lymph nodes) or small bowel dissemination
  - Signs of local irresectability of the primary gastric tumor
  - Recurrent gastric cancer
  - Metachronous peritoneal carcinomatosis
  - Prior resection of the primary gastric tumor
  - Pregnancy, breast feeding, active pregnancy ambition, or unreliable contraceptive methods
  - Uncontrolled infectious disease or known infection with human immunodeficiency virus
  - Known history of hepatitis B or C with active viral replication
  - Recent myocardial infarction (less than 6 months) or unstable angina
  - Uncontrolled diabetes mellitus
  - Any medical condition that is considered to possibly, probably, or definitely interfere with study procedures (including adequate follow-up and compliance) and/or would jeopardize safe treatment
  - Known hypersensitivity for any of the applied chemotherapeutic agents and/or their solvents

### Design

This is a multicenter, open label, phase I-II dose-escalation study. The MTD of docetaxel will be studied using a 3+3 design. The first 3 patients will be treated at the lowest docetaxel dose level (0 mg/m^2^docetaxel), that is, with the fixed dose of oxaliplatin only (460 mg/m^2^). If none of the patients in this dosage cohort experience a dose-limiting toxicity (DLT), the next 3 patients will be treated with a higher docetaxel dose. Docetaxel dosages will be escalated per cohort of at least 3 patients (0, 50, 75, 100, 125, 150 mg/m^2^). If at least 1 of the 3 patients in a dosage cohort experiences a DLT, a total of 6 patients will be treated at the same dose level. When dose-limiting toxicities occur in 2 or more patients in a cohort, no further dose escalation steps will be undertaken (see safety paragraph). The MTD of docetaxel is defined as the dose below the dose level that caused DLT in 2 or more patients in a cohort.

### Sample Size

The sample size cannot be determined upfront since the number of patients will depend on the number of dose-escalation steps. It is expected that 20 to 30 patients will be included in the study.

### Study Procedures

**Figure 1:**
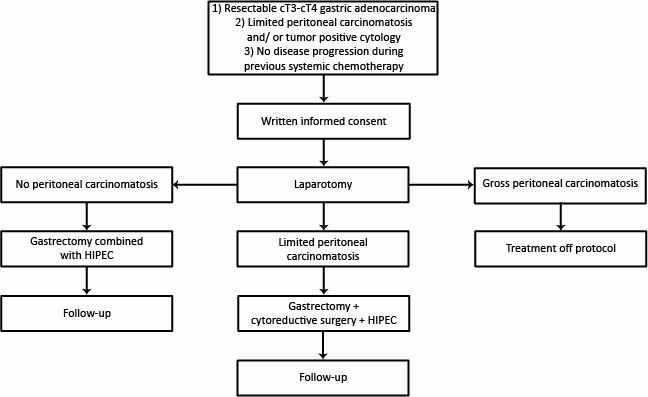
Flowchart of the study design.

Patients with locally advanced (cT3-cT4, any N) gastric adenocarcinoma are eligible for inclusion if the primary gastric tumor is considered resectable, tumor positive peritoneal cytology and/or limited peritoneal carcinomatosis is confirmed by diagnostic laparoscopy/ laparotomy, there is no evidence for distant metastasis, and systemic chemotherapy was without disease progression. Following informed consent, patients will proceed to surgery no longer than 12 weeks after the last course of chemotherapy. The flowchart of this study is shown in Figure 1.

#### Laparotomy

**Figure 2:**
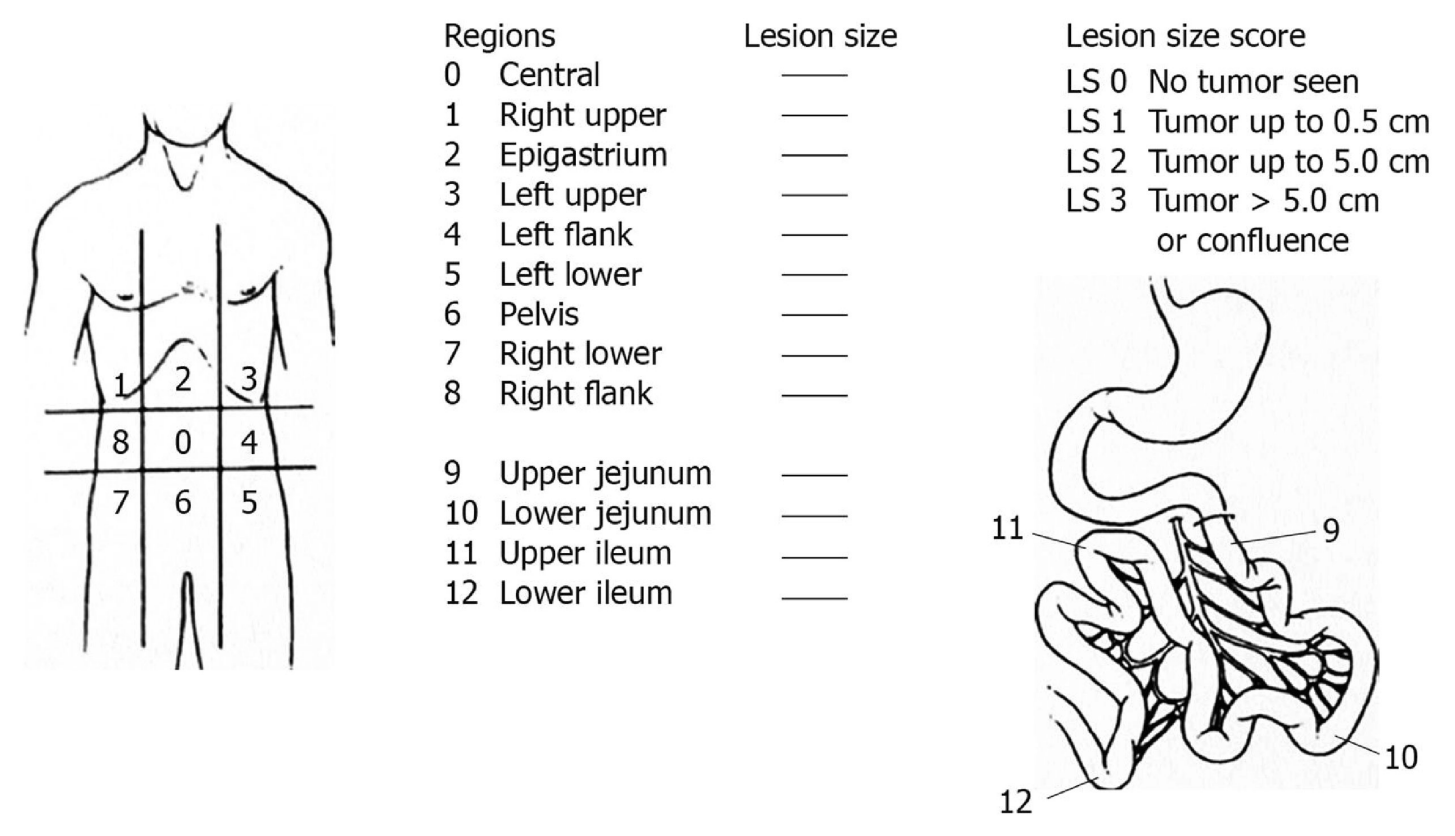
Peritoneal cancer index.

**Figure 3:**
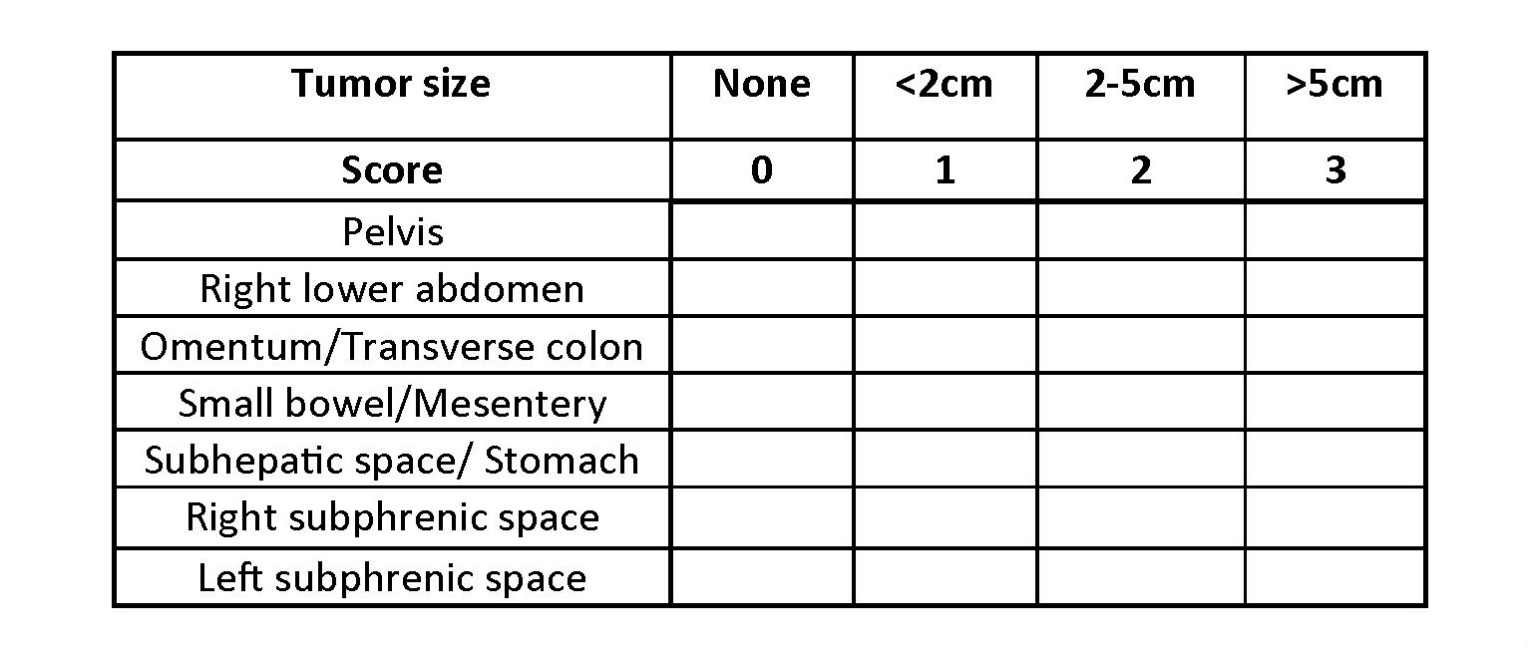
Simplified peritoneal cancer index.

At laparotomy, a thorough inspection of the peritoneal cavity is performed. Before manipulation, the presence and extent of peritoneal tumor deposits will be recorded according to the peritoneal cancer index and the simplified peritoneal cancer index (Figures 2 and 3) [12,13]. Gross peritoneal carcinomatosis (more than one location below the transverse colon and/or small bowel dissemination) is considered tumor progression and will preclude further study participation. Similarly, if a potentially curative gastric resection is not possible, the patient is further treated off study. In these instances, HIPEC is not performed and it will be up to the surgeon to decide on the best palliative surgical intervention (if possible).

#### Gastrectomy, Cytoreductive Surgery, and Hyperthermic Intraperitoneal Chemotherapy

When a potentially curative resection of the primary tumor can be achieved, a total or partial gastrectomy with a D2 lymph node dissection is performed. In patients with limited peritoneal carcinomatosis, cytoreductive surgery will be performed with the objective to leave no macroscopic tumor behind. Peritonectomy is performed as described previously [14]. Reconstruction of the gastrointestinal continuity is postponed until the intraperitoneal chemoperfusion is completed.

HIPEC is performed using an open abdominal technique with 3 inflow and 2 outflow catheters under continuous circulation. The peritoneal cavity is perfused with 460 mg/m^2^oxaliplatin at an intraperitoneal temperature of 41°C to 42°C. After 30 minutes, the perfusion fluid is drained from the abdomen and the peritoneal cavity is perfused with docetaxel for 90 minutes at 37°C. In successive patient cohorts, escalating docetaxel doses will be used (0, 50, 75, 100, 125, 150 mg/m^2^). Gastrointestinal continuity is restored by either a Billroth II or Roux-en-Y reconstruction. A feeding jejunostomy catheter is inserted and will remain in situ until oral intake is adequate.

#### Pharmacokinetics

For pharmacokinetic analysis, plasma and perfusion samples will be obtained at the start, after 15 minutes, and at end of oxaliplatin perfusion, and at the start of docetaxel perfusion, after 45 minutes, and at the end of docetaxel perfusion. Approximately 2 and 12 hours after the operation, plasma samples will be collected.

#### Adjuvant Treatment

Adjuvant treatment is not part of the standard study protocol but it will be discussed for all study patients in the multidisciplinary tumor board meeting. The decision will be made based upon the patient’s individual intraoperative and pathological results, response to previous systemic therapy and experienced toxicity, as well as postoperative recovery.

#### Follow-Up

All patients will be seen at the outpatient clinic once every 3 months for the first year (including the first month after surgery), and every 6 months thereafter until 2 years after surgery. Survival status and disease recurrence/progression will be assessed until death. Follow-up will consist of physical examination, diagnostic investigations (blood tests, computed tomography scans), and hospital admission details (if applicable).

### Safety

There will be at least a period of 2 weeks between the HIPEC procedures within 1 dose-level cohort. To allow adequate toxicity assessment, dose-escalation cannot take place until 4 weeks have elapsed since the last patient's HIPEC procedure in a previous dose level. When the treatment of 3 patients in 1 dose level is completed, the study team will discuss the toxicity and morbidity of the patients in the cohort and will decide in consensus whether dose-escalation can be performed or whether the endpoint of the study has been reached. Toxicity will be graded using the National Cancer Institute (NCI) Common Toxicity Criteria version 4.0. In this study, dose-limiting toxicities include the following events within 30 days after the HIPEC procedure: (1) thrombocytopenia with platelets less than 25.000 x10^6^/L of any duration; (2) grade 4 neutropenia during 7 days or more; (3) grade 3 or 4 febrile neutropenia; (5) grade 3 or higher non-hematological toxicities (excluding grade 3 diarrhea, nausea, vomiting, fatigue, or lethargy); and (6) any other (non)-hematological toxicity, which is regarded as dose-limiting by the investigators.

### Early Stopping Rules

If DLT is observed in more than 1 patient who is treated at the lowest dose level, the current study protocol is considered unsafe and the study will be terminated. At the following dose levels, no further dose escalation steps will be undertaken if DLT occurs in 2 or more patients. In addition, the study team can decide that continuation is undesirable or unethical for other reasons than mentioned in the protocol and thus terminate the study.

### Analysis

Study outcome parameters will be analyzed using descriptive statistical methods. For the calculation of pharmacokinetic parameters, non-compartmental methods will be used. Disease-free and overall survival analyses will be performed by the Kaplan-Meier method for all patients. In these analyses, survival will be measured from the date of start of systemic chemotherapy to the date of disease recurrence and/or death.

### Ethical Considerations

The study protocol has been approved by the medical ethical committee of the Netherlands Cancer Institute/Antoni van Leeuwenhoek Hospital. The study will be performed in accordance with the declaration of Helsinki. The protocol of this study is registered at the Netherlands Trial Registration (NTR) with code NTR4250, and in the Dutch Central Committee on Research Involving Human Subjects (NL42799.031.13). After explanation of the study objectives and procedures (both verbally and in writing), written informed consent will be acquired from all patients.

## Results

Patient recruitment started in January 2014. At first, systemic chemotherapy was part of the study procedure (ie, patients were included prior to chemotherapy). This turned out to hamper patient accrual because most patients were referred after or during systemic chemotherapy given in another hospital. In June 2015, an amendment was submitted in which systemic chemotherapy was abandoned as study treatment (the current protocol). To date, 17 patients underwent the intervention under study (ie, gastrectomy combined with cytoreductive surgery and HIPEC). The results of this study are expected in 2017.

## Discussion

### Study Rationale

The PERISCOPE study aims to determine the safety, tolerability, and feasibility of gastrectomy combined with cytoreductive surgery and HIPEC using oxaliplatin in combination with docetaxel after systemic chemotherapy, as a primary treatment option for patients with advanced gastric cancer with tumor positive peritoneal cytology and/or limited peritoneal carcinomatosis. Previous studies of intraperitoneal chemotherapy in gastric cancer patients suggest that intraperitoneal chemotherapy may be beneficial in selected patients [9,15-17]. However, many of these studies are of limited quality as they were based on heterogeneous patient populations including either patients at risk, being treated prophylactically, or patients with manifested peritonitis treated with therapeutic intent. Most studies use different (neo)adjuvant and intraperitoneal chemotherapy regimens, and many of these studies have been performed in Asian countries which raises the question whether the results can be extrapolated to other ethnic populations.

Well-designed prospective randomized trials are warranted with clearly defined inclusion criteria, distinct neoadjuvant and adjuvant treatment protocols, and with uniform surgical and HIPEC treatment. Prior to such trials, pharmacokinetic studies are mandatory to identify the most optimal chemotherapeutic regimen to be compared to the best available standard treatment. An important issue in intraperitoneal chemotherapy in gastric cancer, therefore, is the choice and dosing of the chemotherapeutic agent. The present PERISCOPE study was designed as the first Western dose escalation trial of intraperitoneal docetaxel in patients with gastric cancer.

### Choice of Intraperitoneal Drugs

Several regimens of intraoperative hyperthermic chemoperfusion in gastric cancer have been explored or are still under investigation [9]. Mitomycin and cisplatin are the most frequently used chemotherapeutic agents, originating from the widespread usage of these drugs in ovarian and colorectal HIPEC. We performed an extensive literature review on the selection of intraperitoneal chemotherapeutic drugs for the use in patients with gastric cancer [10]. Theoretically, a combination of drugs results in more effective treatment. Based on a pioneer study by Elias et al, oxaliplatin is increasingly used as a drug for intraperitoneal chemotherapy with promising results [18]. Oxaliplatin was preferred over cisplatin, as oxaliplatin is not nephrotoxic and appears to have a more favorable pharmacokinetic profile. Following the study by Elias et al, the current dosage of intraperitoneal oxaliplatin is known and widely applied, which provides a valuable starting point for further exploration of combinations of intraperitoneal chemotherapeutic drugs. The taxanes docetaxel and paclitaxel, both seem like promising drugs for intraperitoneal chemotherapy as their systemic uptake is limited, permitting the use of high local concentrations. As the tumor and cell penetration appears to be significantly higher in docetaxel compared to paclitaxel following intraperitoneal administration, we selected docetaxel as the taxane [19]. In addition, severe anaphylactic hypersensitivity reactions have been described of paclitaxel’s solvent Cremophor EL [20]. A combination of oxaliplatin and docetaxel may result in a promising chemotherapeutic regimen. However, no dose-finding study has been performed on intraperitoneal docetaxel administration. Furthermore, the intraperitoneal administration of the combination of oxaliplatin and docetaxel has not yet been investigated in Western patients.

### Patient Selection

In patients with limited peritoneal carcinomatosis and/or tumor positive peritoneal cytology, cytoreductive surgery combined with HIPEC might improve the overall and disease free survival based on current literature. Therefore, this study was considered ethically justified for this patient group. In patients with limited peritoneal dissemination, a complete cytoreduction (ie, complete removal of all macroscopically visible tumor deposits) can be achieved. Complete cytoreduction is one of the key factors associated with improved survival following HIPEC treatment [21,22]. Patients with extensive disease, unresectable tumors, or distant metastases are excluded as the prognosis of these patients is extremely poor. These patients qualify for palliative treatment, or best supportive care only, and are not eligible for an extensive treatment of which the associated complications do not outweigh the potential benefits.

Patients with tumor positive cytology of the peritoneal fluid without macroscopic peritoneal carcinomatosis have a median survival of 15 months and a 5-year survival rate of 0%.[23] As many as 15% of patients without visible metastatic disease will have tumor positive peritoneal cytology, and this proportion will increase to 30% to 50% in patients with serosa-infiltrating tumors or lymph node metastases [24-26]. In these high risk patients, HIPEC is expected to decrease the risk of peritoneal dissemination. Therefore, this group of patients was also included in the study.

### Systemic Chemotherapy

Perioperative treatment has demonstrated an improved progression-free and overall survival in patients with resectable adenocarcinoma of the stomach [1]. Commonly applied regimes include epirubicin with cisplatin and continuous 5-FU (ECF), ECC, and DCF. Similarly, these regimens are used in the palliative setting for metastatic gastric cancer. All 3 regimens have fairly good response rates of 35% to 50%, but the median survival will only be prolonged by a few months and does not surpass 12 months [27]. At present, neoadjuvant chemotherapy is considered standard treatment prior to surgery with curative intent for gastric cancer. In the current study, it was decided that patients prior to inclusion have to be treated with preferably 3 to 4 courses of systemic chemotherapy. All regimens, consisting of a platinum drug combined with a fluoropyrimidine, or those with an additional anthracycline or taxane (according to the local protocol), are accepted. Patients showing progression under systemic chemotherapy were excluded for the study.

### Conclusions

The PERISCOPE study will determine the safety, tolerability, and feasibility of gastrectomy combined with cytoreductive surgery and HIPEC using oxaliplatin in combination with docetaxel after systemic chemotherapy as a primary treatment option for patients with advanced gastric cancer with tumor positive peritoneal cytology and/or limited peritoneal carcinomatosis. The study will provide pharmacokinetic data on the intraperitoneal administration of both oxaliplatin and docetaxel. The acquired study results are a prerequisite for the safe conduct of future studies on HIPEC in patients with gastric cancer, either in the prophylactic or therapeutic setting. The ultimate goal of this ongoing project is to establish a new treatment standard for advanced gastric cancer patients by providing significant survival benefit with acceptable treatment related morbidity.

## Abbreviations

5-FU: 5'fluorouracil
DCF: docetaxel with cisplatin and 5-fluorouracil
DLT: dose limiting toxicity
ECC: epirucibin with cisplatin and capecitabine
HIPEC: hyperthermic intraperitoneal chemotherapy
MTD: maximum tolerated dose
ULN: upper limit of normal
